# PROGgene: gene expression based survival analysis web application for multiple cancers

**DOI:** 10.1186/2043-9113-3-22

**Published:** 2013-10-28

**Authors:** Chirayu Pankaj Goswami, Harikrishna Nakshatri

**Affiliations:** 1 Thomas Jefferson University Hospitals, 117 S 11th Street, Suite 207, Philadelphia, PA 19107, USA; 2 Departments of Surgery, Biochemistry and Molecular Biology, Indiana University School of Medicine, Indianapolis, IN 46202, USA

**Keywords:** Biomarker, Multiple cancer, Survival, Pan cancer, Prognostic, mRNA, Database, Kaplan, Meier, KM

## Abstract

**Background:**

Identification of prognostic mRNA biomarkers has been done for various cancer types. The data that are published from such studies are archived in public repositories. There are hundreds of such datasets available for multiple cancer types in public repositories. Wealth of such data can be utilized to study prognostic implications of mRNA in different cancers as well as in different populations or subtypes of same cancer.

**Description:**

We have created a web application that can be used for studying prognostic implications of mRNA biomarkers in a variety of cancers. We have compiled data from public repositories such as GEO, EBI Array Express and The Cancer Genome Atlas for creating this tool. With 64 patient series from 18 cancer types in our database, this tool provides the most comprehensive resource available for survival analysis to date. The tool is called PROGgene and it is available at http://www.compbio.iupui.edu/proggene.

**Conclusions:**

We present this tool as a hypothesis generation tool for researchers to identify potential prognostic mRNA biomarkers to follow up with further research. For this reason, we have kept the web application very simple and straightforward. We believe this tool will be useful in accelerating biomarker discovery in cancer and quickly providing results that may indicate disease-specific prognostic value of specific biomarkers.

## Background

With advent of high throughput transcriptomic profiling, biomarker identification has been taken to the genomic level. Several studies have been published so far where transcriptomic profiling and consequently biomarker identification in form of single genes, or a signature composed of several genes, has been done on cancer samples, and such data are available in public domain. Gene signatures prognostic for overall, metastasis free or recurrence free survival have been developed using transcriptomic profiling. In several such studies gene signatures have been developed specific for prognostication in particular subtype of a cancer, for instance, a subgroup of population treated with a specific drug. 70 Gene signature Mammaprint® [1], PAM50 [2], OncotypeDx® [3] are some examples of gene signatures of prognostic importance in breast cancer. Similar signatures have also been developed in other cancers such as Colon cancer [4,5], Liver cancer [6], Lung cancer [7,8] and Pancreatic Cancer [9] etc. In any case, the primary endpoint of prognostic assessment is survival analysis, and patient groups are divided into good and bad prognosis groups based on weighted or un-weighted expression of individual genes or a group of genes. Although multiple genes (signatures) provide a stronger and more reliable prognostic assessment, prognostic effects must be first studied at individual gene level. Such an analysis provides rationale for mechanistic studies followed by therapeutic targeting.

Data pertaining to several cancer studies are available in public domain. The wealth of data that is available can be utilized to perform comparative prognostic biomarker identification in multiple cancers. Biomarkers identified using such data as prognostic for one cancer type can also be studied in other cancer types. As mentioned previously, in several studies, biomarkers have been identified for specific populations; however, tools to expand these biomarker sets across multiple cancer types are very limited. Moreover, human genome contains isoforms for several genes that have redundant and non-redundant functions. For example, there are three isoforms for the serine/threonine kinase (AKT) namely AKT1, AKT2, and AKT3. These isoforms have opposing role in cancer or being active only in a specific subtype of cancer. AKT1 promotes tumor growth but inhibits metastasis, whereas AKT2 promotes metastasis [10,11]. Neuronal cell type enriched AKT3 is over expressed in estrogen receptor (ER) negative breast cancer and is a target of frequent translocation in ER-negative but not ER + breast cancer [12,13]. Since AKT is activated in 50% of cancers, it is critical to determine the ratio between these isoforms to generate hypothesis regarding the impact of AKT activation on the course of the disease. However, tools that can analyze data for such purposes are not currently available.

In this paper we present a web tool for identifying prognostic biomarkers in several cancer types. The tool is called 'PROGgene’ and is available at http://www.compbio.iupui.edu/proggene. Our tool can be used to create prognostic (Kaplan-Meier, KM) plots for mRNAs of interest using data in different cancers. To create this tool we have compiled publicly available data from repositories such as Gene Expression Omnibus (GEO), EBI Array Express and recently developed 'The Cancer Genome Atlas’ (TCGA). With a total of 64 datasets from 18 cancer types, our tool is the most comprehensive prognostic biomarker identification tool to date. Currently tools are available to perform prognostic analysis on gene expression data coming from public domain, e.g., KMplot for Breast [14] and Ovarian [15] cancer, and ITTACA [16] and Prognoscan [17] for multiple cancers. But these tools suffer from some limitations which are overcome in our tool as discussed in the sections ahead. Using PROGgene, users can also divide data into several subgroups based on covariates available for dataset of interest. For e.g., prognostic plots can be created by dividing the patient series into groups of patients treated and not treated with chemotherapy, or groups at different stages of disease. The web application provides a list of datasets available for analysis of interest and lets researcher choose the most pertinent datasets for their study design. Researchers may look for more information on datasets providing promising plots in the source repositories with links provided on the results page to further identifying the study characteristics they are looking for.

We believe that this tool would prove to be an important hypothesis generation tool for researchers working in area of cancer. Since more and more datasets are being continually uploaded in the public repositories, we would survey the public repositories periodically for newly uploaded data to extend the repository of datasets. We are not planning to implement any automated data retrieval tool from these repositories to deposit new data in our database. Rather we would manually curate and process the data before it is available in our dataset for the reason that every dataset requires different parameters for processing. In planned future version of our tool, we would also like to produce more informative graphs by introducing adjustments for survival covariates in survival analysis. Researchers who cannot see datasets of their interest in our repository can also request addition of datasets using contact information provided on the website.

## Construction and content

### Data

**Figure 1:**
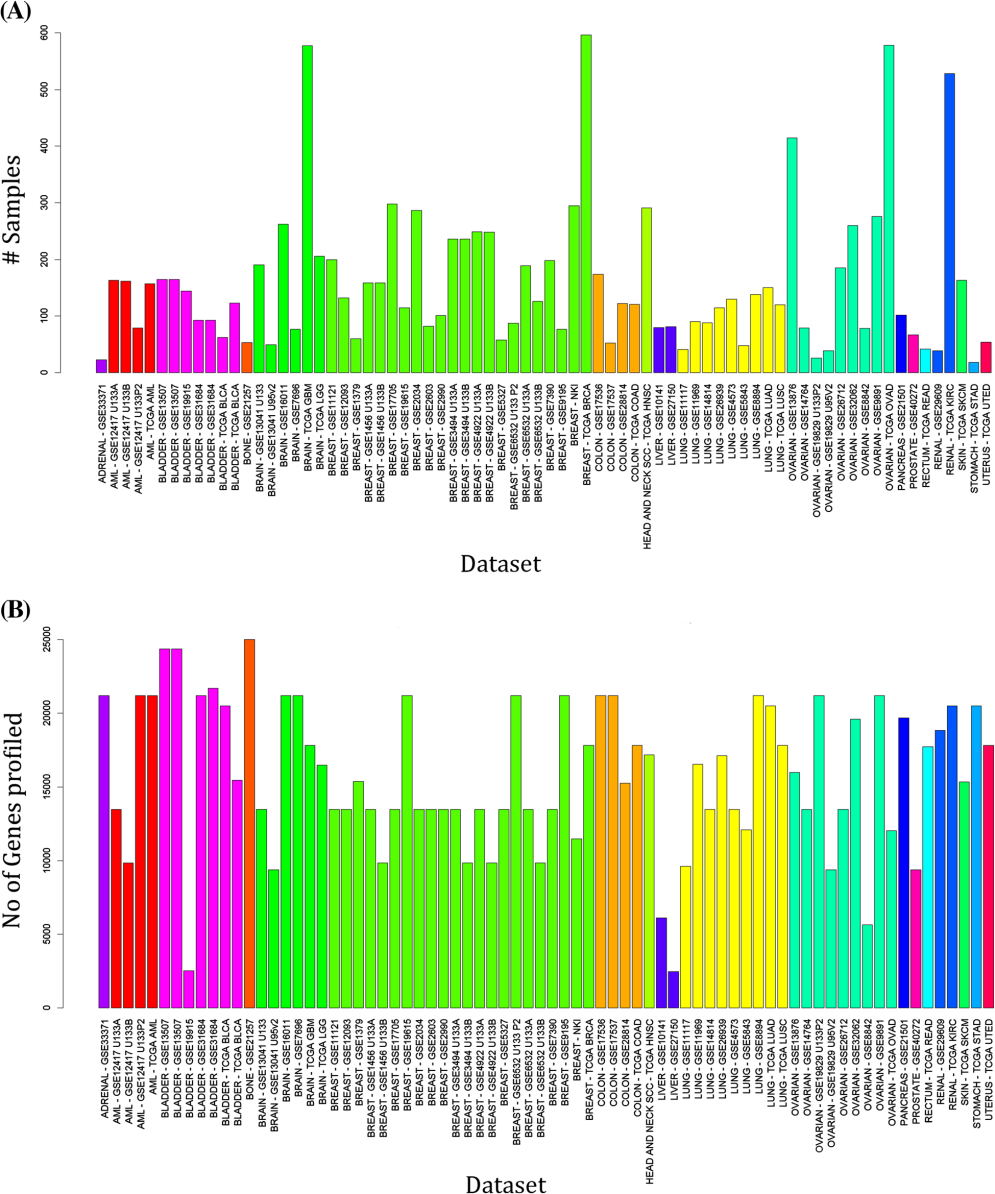
**Descriptive statistics on datasets included in PROGgene database. (A)** No of samples per dataset available in PROGgene. **(B)** No of genes profiled in each dataset available in PROGgene.

We have compiled data on 64 patient series’ in our database. Additional file 1: Table S1 lists the datasets available in our database with information on data source, platform of transcriptomic profiling, number of samples (after screening off samples that did not have relevant survival related information) in the series and number of genes available. A distribution of samples and genes in the database is also provided in Figure 1. In majority of the series, we have downloaded raw data from the public repositories and processed the data ourselves. Data preprocessing was done in following ways

a) For datasets where preprocessed data were downloaded from public repositories (such as series matrices from GEO), no further processing of data was done.

b) For datasets where raw data profiled on Affymetrix arrays was downloaded, data were imported into Partek Genomics Suite (v 6.5) (PGS) using RMA background correction. If batch effect was present in the data due to samples being profiled in separate batches, it was removed using PGS’s batch effect removal tool. Finally, probe level data was collapsed to gene level data by retaining only the probe with maximum coefficient of variation across all samples, and discarding the rest of probes for genes that were profiled on multiple probes.

c) For datasets where raw data profiled on Illumina platform were downloaded, probes that showed insufficient signal and p values across more than 20% of samples were discarded. For the probes that remained, they were collapsed to gene level data in a way similar to that for Affymetrix data.

For all the data, final probability distributions for all samples were checked manually using PGS (data not shown). Datasets that showed an abnormal distribution of genomic profiles were discarded. Additional file 2: Table S2 lists various survival variables associated with available datasets. For all other cancers except breast cancer, we have one or more of Overall, Metastasis free and Recurrence free survival variables. For Breast cancer, apart from overall, metastasis free and recurrance free survival, for some datasets Lung and Brain Metastasis free survival measures are also available. Additional file 3: Table S3 enlists covariates available for the different datasets. Additional file 4: Table S4 enlists for each datasets, number of samples, median and minimum and maximum survival times associated with various survival functions available for the dataset.

### Web application

Our tool is a web application created using PHP5 [18] and R Programming environment (v2.15.2), with MySQL (v 5.0.95) [19] database in the backend. Survival calculations are done in our tool using R library 'Survival’ which is also coded in the backend. The web application consists of Home page where users can input gene(s) of interest and select cancer type in which they want to create prognostic plots. Here users can also select the survival function they want to study such as overall survival or metastasis free survival or recurrence free survival. Upon submitting the information in home page, a list of all datasets which are relevant to the current analysis parameters is displayed. Users can select datasets they want to visualize prognostic plots in from the list on filter page. Upon submitting information on this page, prognostic plots are calculated by backend scripts and results are displayed on the results page. If more than one datasets are available for selected cancer type for selected parameters, prognostic plots are displayed separately for each dataset. If covariates are selected for dividing the data, for each dataset, besides the global plot, prognostic plots are also shown for data divided by covariates. When multiple genes are being studied, the application also produces a plot for average gene expression for full signature. In case of gene signature, data division by covariates is not done, and only global signature plots are provided. The tool also indicates whether expression data for one or more genes in the signature is not available in any dataset.

### Workflow

**Figure 2:**
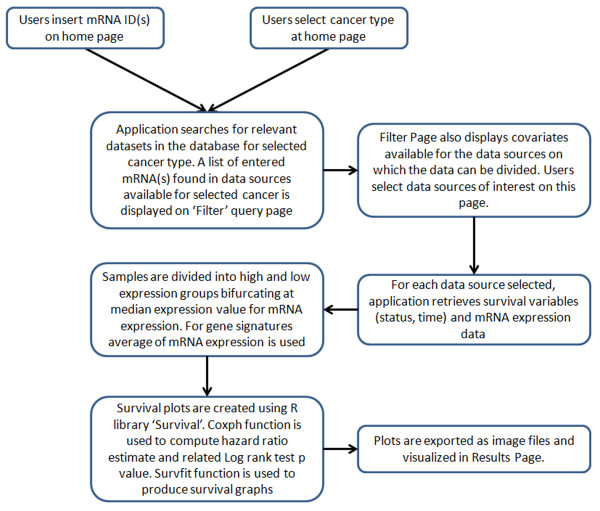
Flowchart depicting workflow of PROGgene program.

To create prognostic plots our tool uses R library 'Survival'. A line diagram depicting workflow of our tool is provided in Figure 2. Although prognostic plots can be created for multiple genes using their average expression in our tool, for the purpose of illustrating methodology, we would explain how prognostic plots are created for a single gene. Users enter gene symbol and select cancer type in the home page of our tool. For selected cancer type, all datasets available are retrieved. In each dataset, for the selected gene, survival information in terms of survival status (overall or metastasis free or recurrence free survival), and survival time (time to death or time to metastasis or time to recurrence) are retrieved along with gene expression as continuous variable. Using median gene expression value as bifurcating point, samples are divided into High and Low gene expression groups. Using survival data and continuous expression variable, survival analysis is done by fitting cox proportional hazards model using function “coxph” of library survival. Hazard ratio (HR) as 'exp(coef)’ and log rank p value are retrieved from the fitted model. To create prognostic plot, High and Low expression categorical variable is used along with survival data. Plots are created using function 'survfit’ of the same R library. Final plots, which show survival in High and Low expression arms of samples, annotated for HR, HR confidence intervals and p value are exported as '.png’ images, which are displayed on the results page.

## Utility and discussion

**Figure 3:**
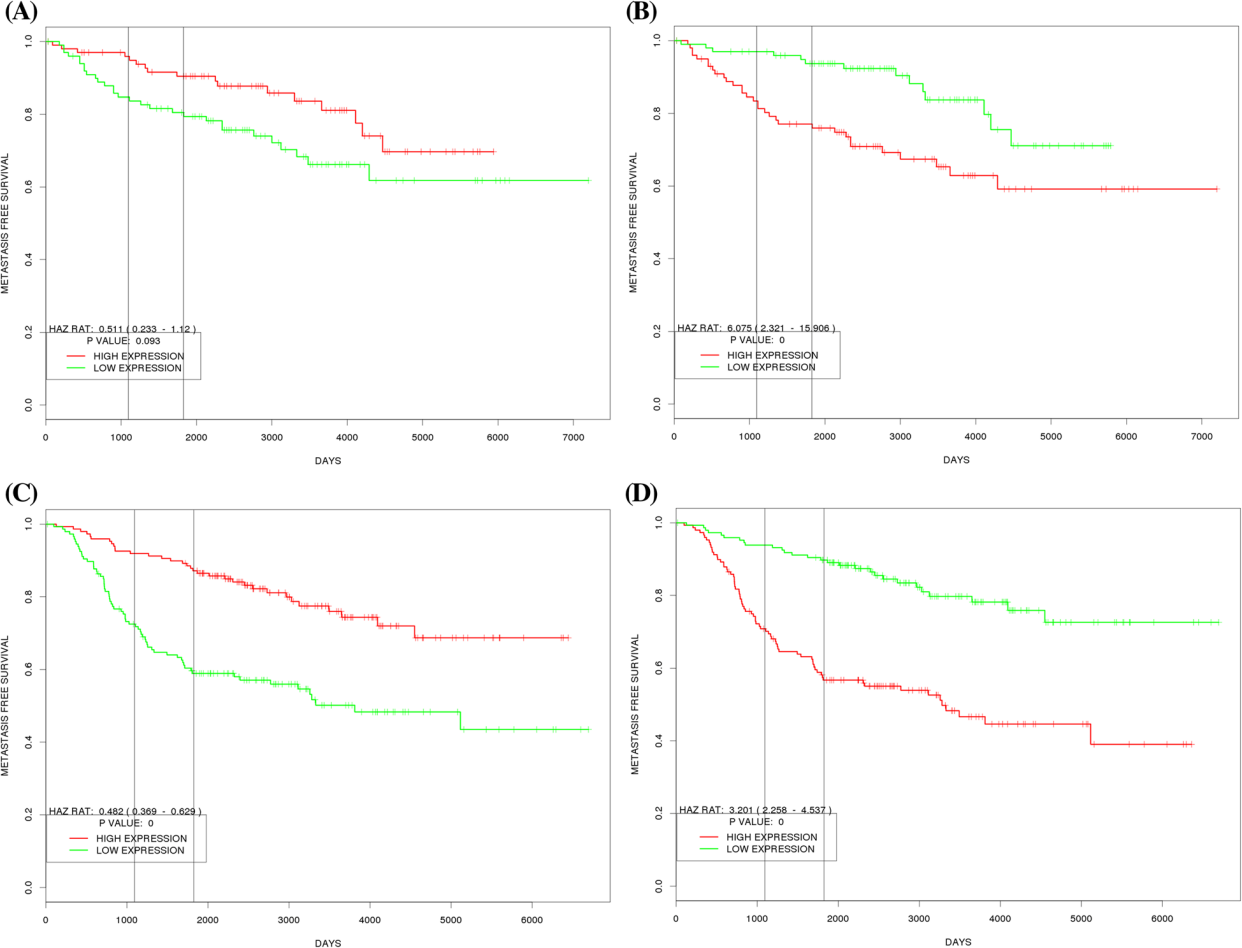
**Prognostic Plots created with PROGgene for published signatures in Breast Cancer. (A)** Combined prognostic plot for genes down regulated in poor prognosis group in 70 gene Mammaprint signature of breast cancer in GSE11121, **(B)** Combined prognostic plot for genes up regulated in poor prognosis group in 70 gene Mammaprint signature of breast cancer in GSE11121, **(C)** Combined prognostic plot for genes down regulated in poor prognosis group in 70 gene Mammaprint signature of breast cancer in NKI dataset, **(D)** Combined prognostic plot for genes up regulated in poor prognosis group in 70 gene Mammaprint signature of breast cancer in NKI dataset.

**Figure 4:**
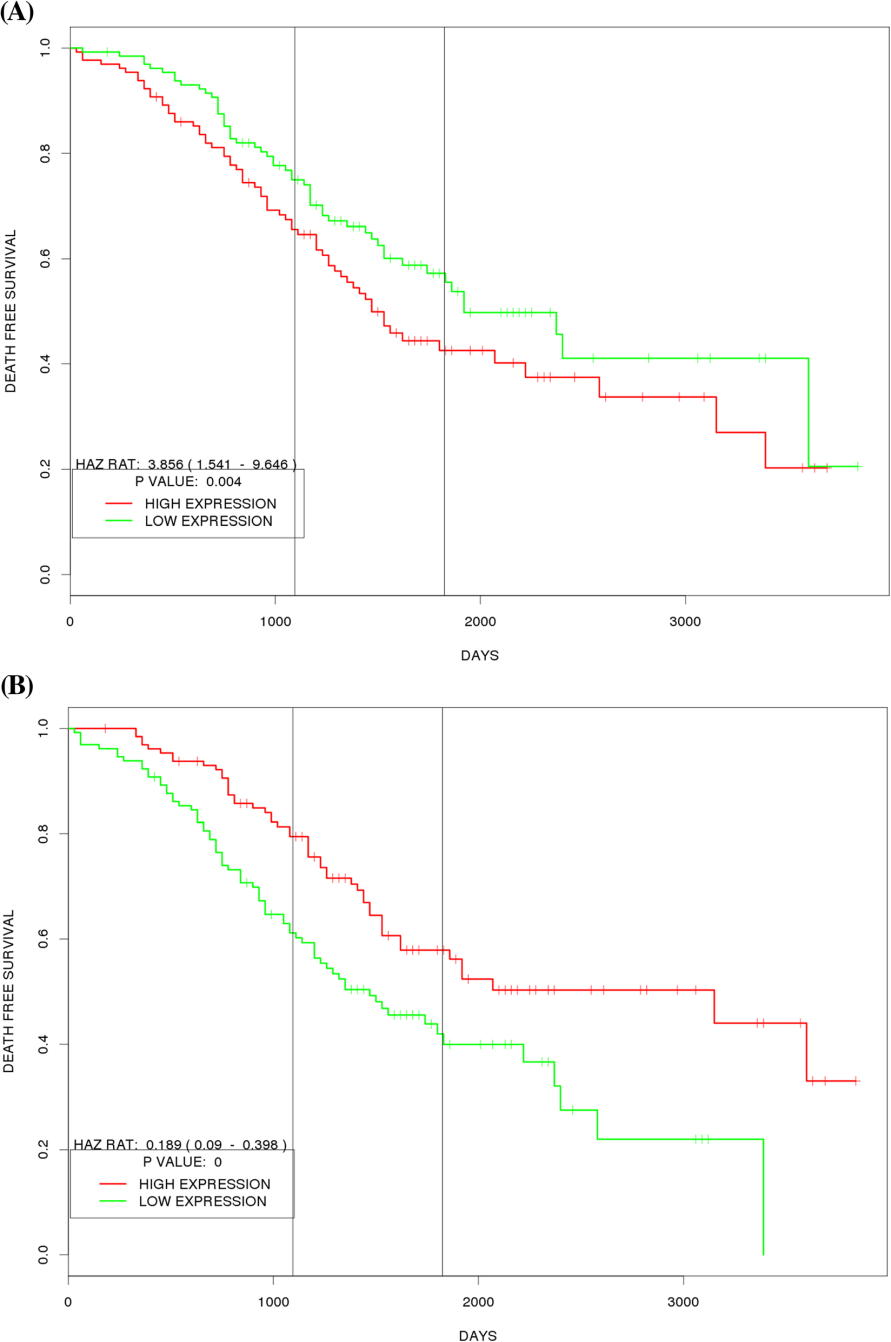
**Prognostic Plots created with PROGgene for published signatures in TCGA Ovarian Cancer. (A)** Combined prognostic plot for genes up regulated in poor prognosis group in 193 gene TCGA signature for Ovarian cancer in GSE32062, **(B)** Combined prognostic plot for genes down regulated in poor prognosis group in 193 gene TCGA signature for Ovarian cancer in GSE32062.

We assessed the performance of our tool by creating prognostic plots for recently published biomarker signatures as case studies using our tool. In majority of cases the prognostic plots created by our tool corroborated with the published findings (data not shown). In first case study, we created prognostic plots for genes associated with poor and good outcomes in the 70 gene Mammaprint® signature for breast cancer. The 70-gene signature was identified in node negative breast cancer samples and predicts outcome as time to metastasis in such patients. Of the 70 genes in the signature roughly 70% genes are over expressed in poor prognosis group while the rest are under expressed in the same group compared to the good prognosis group. In this study we created prognostic plots using our application for good and bad prognosis related genes in Mammaprint signature in two external datasets–NKI dataset [20] and GSE11121 [21]. GSE11121 was chosen as the second dataset as it also consists of node negative tumor samples. Figure 3 shows combined prognostic plots for genes up and down regulated in 70-gene signature separately in the two datasets. Although all the genes in Mammaprint signature were not found in these datasets, the resulting plots for sum of found genes corroborates in external datasets with the findings in the published work. In another case study, we tried to validate the gene signature developed by the Cancer Genome Atlas consortium for predicting overall survival in ovarian cystadenocarcinoma. The TCGA consortium identified a 193 gene signature predictive of overall survival in high stage and grade ovarian cystadenocarcinoma patients. The signature consists of 108 genes associated with poor prognosis and 85 genes associated with good prognosis. We created plots for both subsets of genes in external dataset GSE32062 [22] which comprises of samples from high grade and stage ovarian cancer patients. Some genes from the TCGA signature were not present in this dataset, but the plots for combination of rest of the genes from the two subsets again corroborated with the directions identified in the TCGA publication (Figure 4).

Our tool is primarily a hypothesis generation tool, which is meant to provide pursuable gene biomarkers in cancers of choice. Our database contains data from 64 unique patient series’, amounting to a total of approximately 11,800 samples profiled over a maximum of approximately 24,000 markers in 18 cancer types. Patient samples in the datasets come from a variety of populations and expression profiling platforms. The web application implements traditional survival analysis toolkit using data from public repositories and is thus a pipeline rather than a novel method.

Our tool has significant advantages over other contemporary similar tools. First, with 18 cancer types our tool is the most comprehensive tool to date for survival analysis and can be used by researchers working on a wide array of cancer types. Secondly, our tool does not merge data coming from different studies (having different characteristics) and platforms, which may in certain situations, become erroneous. Third, an indirect advantage of this strategy is that researchers can identify study characteristics where their potential biomarkers may not work at all or may have an inverse effect. Tools like ITTACA are not primarily survival analysis tools and thus, do not have capability of producing survival plots for a lot of different cancer types and studies. ITTACA comprises data for only 7 cancer types and is capable of conducting survival analysis on only a few cancer types using data from only a limited number of studies. PrognoScan although compiles data for 14 cancer types, does not include recent major datasets such as TCGA data, and also cannot be used to study prognostic implications of multiple genes (signatures). KMplot for Breast and Ovarian Cancer suffer from inherent over fitting of data as they normalize gene expression data coming from several different studies and to pool one large patient series, in an attempt to provide meta-analysis. Merging of dataset using currently available algorithms can be performed on datasets profiled only on same platform for optimal results. For this reason, KMplot merges data coming from a single gene expression profiling platform. Although a very robust tool, this strategy in KMplot may lead to misleading results when studying biomarkers identified on other platforms. For instance, Crijns et. al. [23] identified an 86 gene signature predictive of overall survival in high risk (high grade and stage) ovarian cancer patients. Gene expression in this study was profiled on custom microarray platform. We tried to plot prognostic plot for this signature using KMPlot and PROGgene separately. For 60 genes from the signature whose decreased expression is associated with higher risk (low overall survival rate), Affymetrix probe IDs (usable in KMplot) were available for only 30 genes. Using these probe Ids KMplot failed to produce statistically significant KM plot for this group of genes (P > 0.1) using high stage (3 + 4) and grade (3) as study parameters. For the same signature we also performed survival analysis using KMplot for the group of genes whose higher expression is associated with high risk. This analysis also failed to produce statistically significant results. For the same group of genes, using PROGgene we were able to produce a significant prognostic plot using datasets which comprise of gene expression profiling of high stage and grade ovarian cancer patients (GSE32062 and TCGA, see Additional file 5).

In PROGgene we have kept gene expression data coming from same patient series, but profiled on different platforms separate. The motivation behind providing study specific prognostic plots rather than a pooled prognostic plot is because we believe researchers are more interested in analyzing data which is more pertinent to their specific design for hypothesis generation. Datasets which do not contain any information relevant to such specific study designs may lead to irrelevant information. Although, this limits sample size in some series, we believe this approach is highly suitable for hypothesis generation as it provides prognostic plots pertaining to different subpopulations of cancers separately, and does not perform any over-fitting of the data. Another reason for not merging data from different studies is that many of studies have been conducted using microarray platforms and these platforms are becoming obsolete. The focus of Transcriptomic profiling technology is shifting more and more towards sequencing. With evolving sequencing technology it is not possible to merge gene expression profiles profiled in different experiments together.

In future versions of the tool, we would like to add more variables to our database as more and more integrated data repositories such as TCGA evolve. We also plan to incorporate adjusting of survival models for covariates such as age, therapy, sex, hormonal statuses etc., for providing more informative prognostic plots to the researchers. As more and more data becomes available, such incorporations are possible.

## Conclusion

Prognostic biomarker identification, which may include genes, polymorphisms, mutations, micromolecules, or epigenetic regulators, is one of the major contributions of cancer genomics. Cancer research predominantly focuses on specific patient populations for biomarker identification. We believe that this application will prove useful to researchers working on cancers to identify potential gene biomarker targets for hypothesis generation and designing mechanistic studies. Our tool uses data pertaining to specific patient population as published in studies. It allows users to divide data in specific studies by available covariates and analyze different survival functions on such divided data. This allows researchers to study biomarkers in specific patient populations without the problem of over fitting the data. Future version of our tools will also allow researchers to adjust survival models for different covariates and will provide more intuitive plots.

## Availability and requirements

The tool is a web application and is available freely for academic and research purposes at following URL http://www.compbio.iupui.edu/proggene.

## Abbreviations

TCGA: The cancer genome atlas; HR: Hazard ratio; KM: Kaplan meier; PGS: Partek genomics suite.

## Competing interests

The authors declare that they have no competing interests.

## Authors’ contribution

CG carried out study planning, data procurement, quality assurance, data analysis, programming and web development, and manuscript preparation for the article. HN contributed to study planning and manuscript preparation. Both authors read and approved the final manuscript.

## Supplementary Material

Additional file 1: Table S1 Table enlisting various datasets available in PROGgene. Click here for file

Additional file 2: Table S2 Table enlisting various survival measures available for the datasets available in PROGgene. Click here for file

Additional file 3: Table S3 Table enlisting the covariates associated with the datasets available in PROGgene. These covariates can be used to divide data for creating prognostic plots for subpopulations within one dataset. Click here for file

Additional file 4: Table S4 Table enlisting descriptive statistics such as sample size, max, median and min survival times associated with datasets available in PROGgene. Click here for file

Additional file 5 Prognostic plots created using KMPlot and PROGgene for gene signature identified as predictive of high risk (overall survival) in ovarian cancer by Crijns et. al. Click here for file
