# Supplementary material for: PROGgene: gene expression based survival analysis web application for multiple cancers
Source: J Clin Bioinforma. 2013 Oct 28;3:22. doi: 10.1186/2043-9113-3-22 (PMC3875898; doi:10.1186/2043-9113-3-22)

Supplementary Information

Prognostic plots created using KMPlot and PROGgene for gene signature identified as predictive of high risk (overall survival) in ovarian cancer by Crijns et. al.

Crijns et. al. identified a 86 gene signature predictive of overall survival in high risk ovarian cancer patients. The gene signature identified 60 markers for which downregulated expression and 26 markers for which up-regulated expression is associated with high risk in ovarian cancer patients. We conducted survival analysis for the gene signature whose down regulation is associated with bad prognosis using PROGgene and KMPlotter. Since KMPlotter uses only Affymetrix IDs, we identified Affymetrix IDs associated with the genes in the signature. For 30 genes affyemtrix IDs were obtained. The list of genes, their association with overall survival and associated probe IDs is listed in the following table

Table: Gene signature, trend of expression of individual genes, risk association and associated Affymetrix IDs for gene signature identified as prognostic in Ovarian Cancer by Crijns et. al.

Gene Symbol	Expression in High risk group	Risk	Probe Set ID
KIAA0141	DOWN	LOW	201978_s_at
DDB2	DOWN	LOW	203409_at
GNAZ	DOWN	LOW	204993_at
FGFBP1	DOWN	LOW	205014_at
RIN1	DOWN	LOW	205211_s_at
ITGB7	DOWN	LOW	205718_at
CNTFR	DOWN	LOW	205723_at
JAK2	DOWN	LOW	205842_s_at
CRYBB1	DOWN	LOW	206185_at
GCM1	DOWN	LOW	206269_at
MXD1	DOWN	LOW	206877_at
CDH19	DOWN	LOW	206898_at
CACNA1B	DOWN	LOW	207162_s_at

MUTYH	DOWN	LOW	207727_s_at
PTPRN2	DOWN	LOW	211534_x_at
BAX	DOWN	LOW	211833_s_at
HIPK1	DOWN	LOW	212293_at
ATP5D	DOWN	LOW	213041_s_at
CES2	DOWN	LOW	213509_x_at
OSM	DOWN	LOW	214637_at
AAK1	DOWN	LOW	214998_at
ACSM1	DOWN	LOW	215432_at
C1orf68	DOWN	LOW	217087_at
KYNU	DOWN	LOW	217388_s_at
PPCDC	DOWN	LOW	219066_at
C1orf159	DOWN	LOW	219337_at
METTL4	DOWN	LOW	219698_s_at
AIPL1	DOWN	LOW	219977_at
NCR2	DOWN	LOW	221075_s_at
PTCH2	DOWN	LOW	221292_at
KDELR2	UP	HIGH	200700_s_at
CPE	UP	HIGH	201117_s_at
MFAP2	UP	HIGH	203417_at
LRRC17	UP	HIGH	205381_at
FEZ1	UP	HIGH	205973_at
RIT1	UP	HIGH	209882_at
PPAP2B	UP	HIGH	212230_at
KRT10	UP	HIGH	213287_s_at
KLF12	UP	HIGH	214276_at
KLHL7	UP	HIGH	220239_at
OSGEPL1	UP	HIGH	220631_at
C1QTNF3	UP	HIGH	220988_s_at
PRELP	UP	HIGH	37022_at

We used the gene lists to perform survival analysis using KMPlot in high grade (3) and stage (3+4) ovarian cancer patients. KMPlot produced insignificant prognostic plots for the mean expression of these probe groups.

Fig 1: KMPlot output for HIGH risk group genes

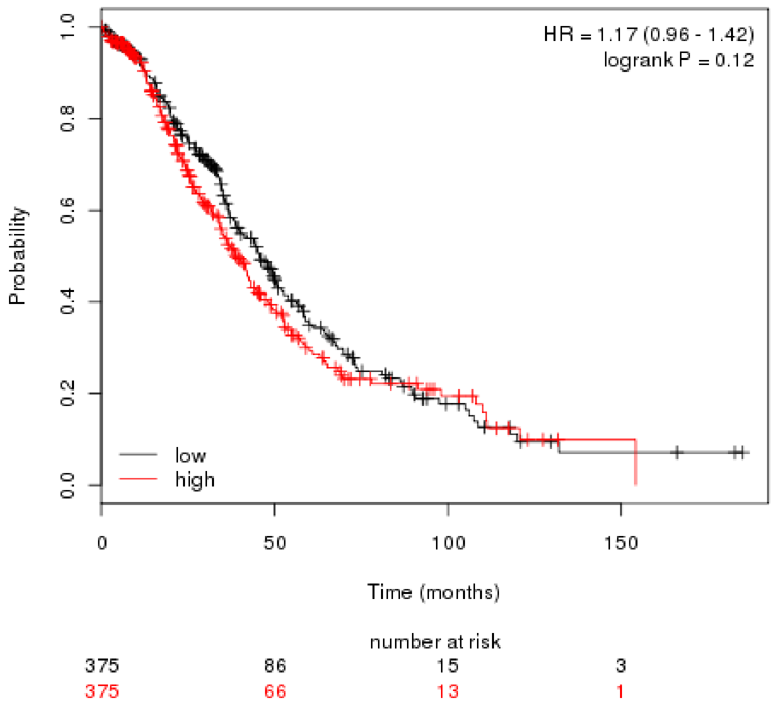

Fig 1: KMPlot output for LOW risk group genes

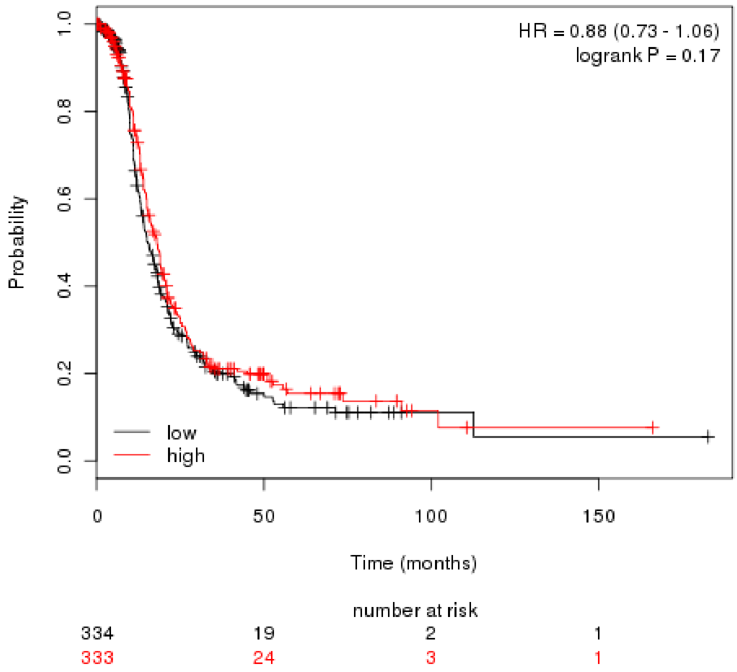

Fig 3: Prognostic plot create for LOW risk group genes using ProgGene in GSE32062

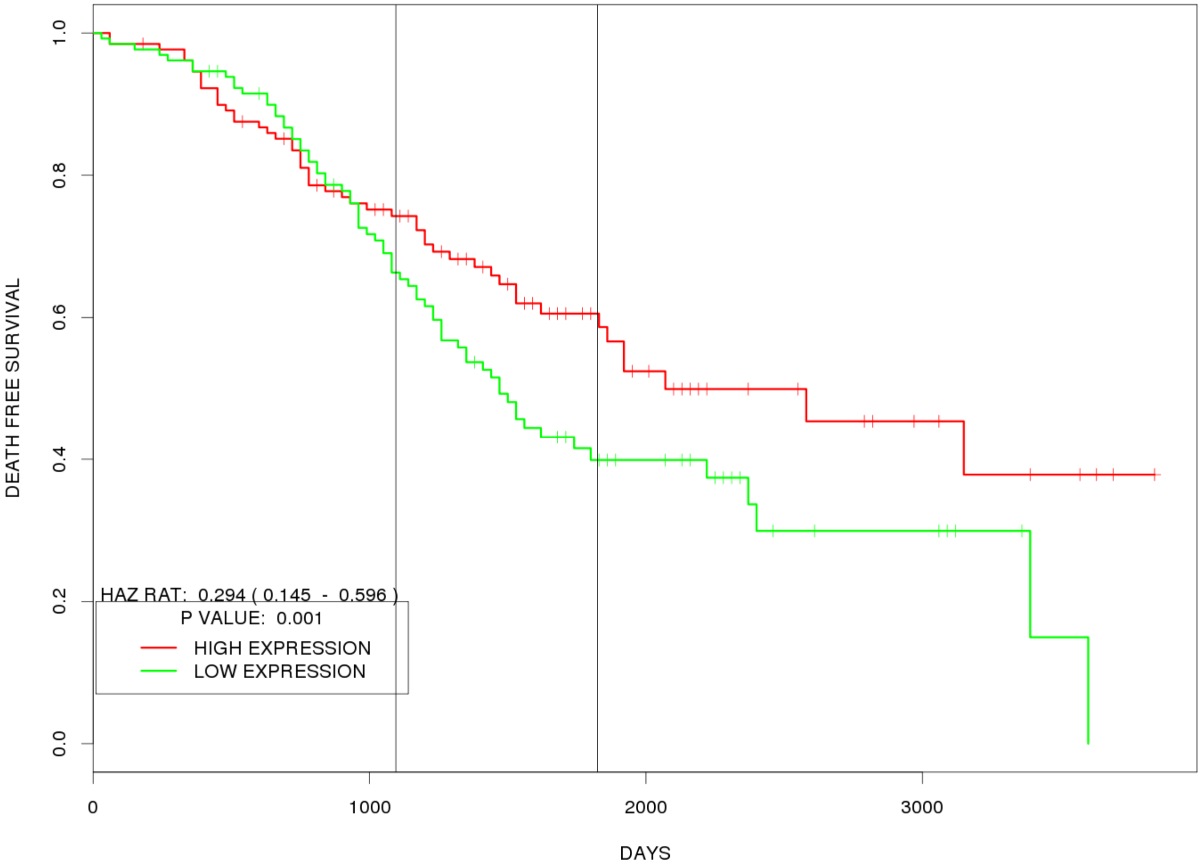

GSE32062 - Immune-activation as a therapeutic direction for patients with high-risk ovarian cancer based on gene expression signature

Fig 3: Prognostic plot create for LOW risk group genes using ProgGene in TCGA

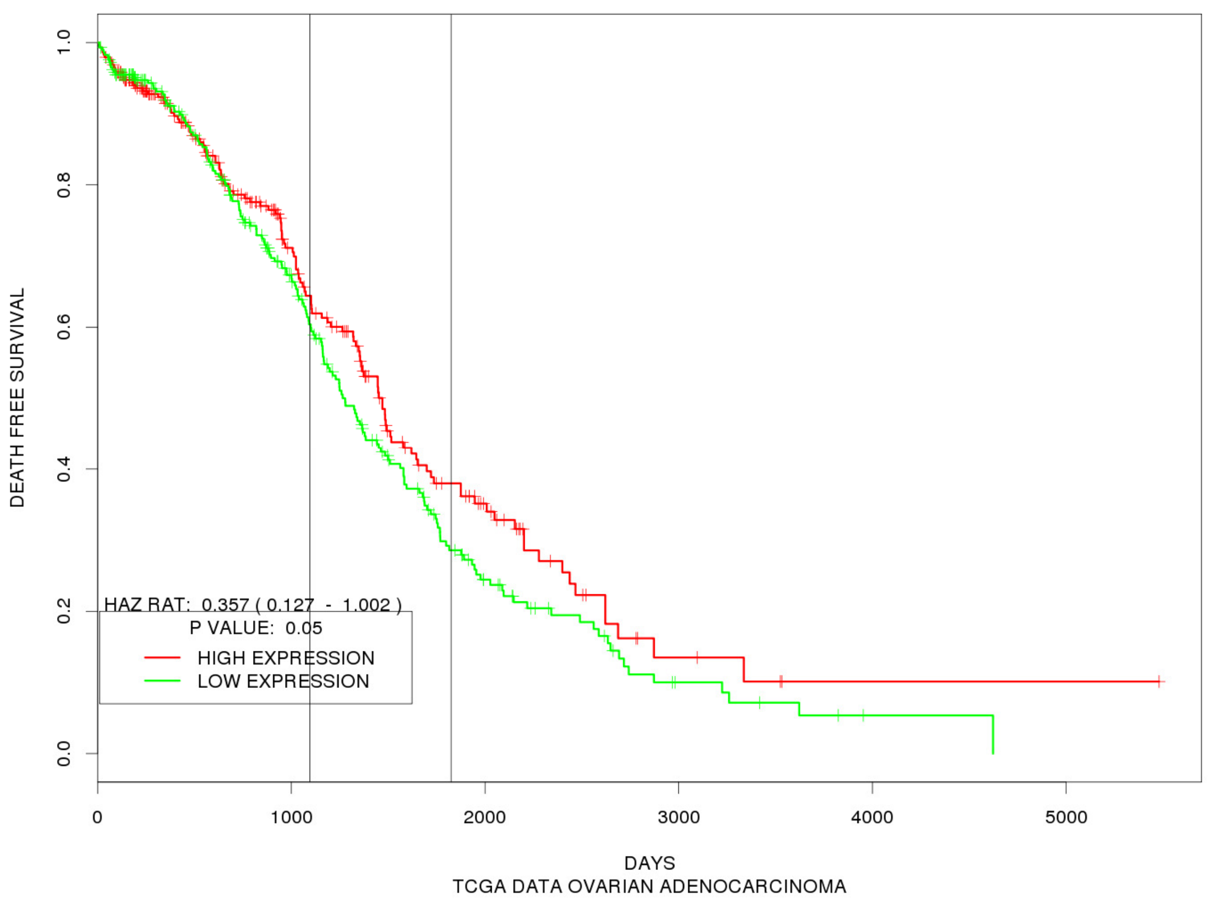

Supplement: Additional file 5 — Prognostic plots created using KMPlot and PROGgene for gene signature identified as predictive of high risk (overall survival) in ovarian cancer by Crijns et. al. [file 2043-9113-3-22-S5.pdf]
